# The RNA-Binding Protein ESRP1 Modulates the Expression of RAC1b in Colorectal Cancer Cells

**DOI:** 10.3390/cancers13164092

**Published:** 2021-08-13

**Authors:** Marta Manco, Ugo Ala, Daniela Cantarella, Emanuela Tolosano, Enzo Medico, Fiorella Altruda, Sharmila Fagoonee

**Affiliations:** 1 Molecular Biotechnology Center, Department of Molecular Biotechnology and Health Sciences, University of Turin, 10126 Turin, Italy; marta.manco@unito.it (M.M.); emanuela.tolosano@unito.it (E.T.); 2 Department of Veterinary Science, University of Turin, Largo Paolo Braccini 2, 10095 Grugliasco, Italy; ugo.ala@unito.it; 3 Department of Oncology, University of Torino, S.P. 142, km 3.95, Torino, 10060 Candiolo, Italy; daniela.cantarella@ircc.it (D.C.); enzo.medico@ircc.it (E.M.); 4 Institute of Biostructure and Bioimaging, National Research Council (CNR) c/o Molecular Biotechnology Center, 10126 Turin, Italy

**Keywords:** colorectal cancer, ESRP1, RNA-binding protein, *RAC1*, *RAC1b*, alternative splicing, cDNA microarray, bioinformatics analysis

## Abstract

**Simple Summary:**

Colorectal cancer (CRC) ranks third for incidence and second for number of deaths among cancer types worldwide. Poor patient survival due to inadequate response to currently available treatment regimens points to the urgent requirement for personalized therapy in CRC patients. Our aim was to provide mechanistic insights into the pro-tumorigenic role of the RNA-binding protein ESRP1, which is highly expressed in a subset of CRC patients. We show that, in CRC cells, ESRP1 binds to and has the same trend in expression as *RAC1b*, a well-known tumor promoter. Thus, RAC1b may be a potential therapeutic target in ESRP1-overexpressing CRC.

**Abstract:**

RNA binding proteins are well recognized as critical regulators of tumorigenic processes through their capacity to modulate RNA biogenesis, including alternative splicing, RNA stability and mRNA translation. The RNA binding protein Epithelial Splicing Regulatory Protein 1 (ESRP1) can act as a tumor suppressor or promoter in a cell type- and disease context-dependent manner. We have previously shown that elevated expression of ESRP1 in colorectal cancer cells can drive tumor progression. To gain further insights into the pro-tumorigenic mechanism of action of ESRP1, we performed cDNA microarray analysis on two colorectal cells lines modulated for ESRP1 expression. Intriguingly, RAC1b was highly expressed, both at mRNA and protein levels, in ESRP1-overexpressing cells, while the opposite trend was observed in ESRP1-silenced CRC cells. Moreover, *RAC1* and *RAC1b* mRNA co-immunoprecipitate with ESRP1 protein. Silencing of RAC1b expression significantly reduced the number of soft agar colonies formed by ESRP1-overexpressing cells, suggesting that ESRP1 acted, at least partially, through *RAC1b* in its tumor-promoting activities in CRC cells. Thus, our data provide molecular cues on targetable candidates in CRC cases with high ESRP1 expression.

## 1. Introduction

Colorectal cancer (CRC) ranks third for prevalence and second for number of deaths among all cancers worldwide [1]. According to the worldwide cancer burden in 2020 and GLOBOCAN estimates of cancer incidence and mortality produced by the International Agency for Research on Cancer, CRC was fifth among cancers with newly diagnosed cases, as well as deaths in 2020 [1]. Management of this malignant tumor is achieved by several strategies, including chemotherapy and targeted therapy using small molecules [2]. However, poor patient survival, due to inadequate response to currently available treatment regimens and shortage of adequate risk-assessment biomarkers, points out to the urgent requirement for personalized therapy in CRC patients.

In an era of human genomic, transcriptomic and proteomic exploration of human pathologies through high throughput technologies, dissecting the molecular pathways involved in oncogenic processes to design new patient-tailored therapies has become feasible. Several biomolecules work in concert to initiate and promote tumorigenesis. The crucial role of several oncogenic and tumor suppressor transcription factors in CRC initiation and progression have been reported. For instance, the epithelial to mesenchymal transition (EMT)-regulators zinc finger E-box- binding homeobox-(ZEB)-1 and -2, snail family transcriptional repressor 2 (SLUG), snail family transcriptional repressor 1 (SNAI1) and TWIST-related protein 1 (TWIST) are critical drivers of tumor progression [3,4]. Epigenetic changes such as DNA methylation and histone modifications are implicated in several CRC-associated pathways [5]. Non-coding RNAs, including long non-coding RNAs and microRNAs, exert regulatory functions in the multistep processes of carcinogenesis [6,7,8]. RNA binding proteins (RBPs) have also surged as essential modulators in every cancer hallmark, both as tumor promoters and suppressors, as well as regulators of genetic stability. Recently, an integrated analysis of RBPs in human CRC was performed by Fan et al. [9]. The authors identified 12 prognostic RBPs, which were modulated in CRC and warrant in-depth studies. RBPs have pleiotropic activities in RNA processing and metabolism, such as alternative splicing and translation. They can interact with proteins and different types of RNA species to generate ribonuclear protein complexes with different combinations in a context- and disease-specific manner, thus putting RBPs and related pathways at the forefront of cancer therapeutics development [10].

Aberrant expression of RBPs influences cancer-related cellular phenotypes. We have previously shown that the overexpression of Epithelial Splicing Regulatory Protein 1 (ESRP1) can promote cancer traits in CRC cells, by increasing their anchorage-independency and enhancing their clonogenic capacity in vitro, as well as promoting macrometastasis formation in vivo [11]. Autocrine activation of fibroblast growth factor receptor (FGFR2IIIb, a splicing target of ESRP1) played an important part in these phenotypes. There was marked PI3K/AKT pathway activation and SNAI1 protein overexpression, which skewed the cells towards adopting a partially mesenchymal, and more tumorigenic, molecular profile. These data were further supported by proteomics data on ESRP1-modulated CRC cells [12]. The observation that ESRP1 can have pro-tumorigenic and pro-metastatic activities was also shown by other studies [13,14,15]. Leontieva et al. demonstrated that ESRP1 could exert differential effects on protein translation of oncogenic mRNAs, such as *MYC* and *FOS*, according to the complexity of their 5′UTR secondary structure [15]. Yae et al. reported that ESRP1 promotes the splicing of CD44v isoform, which stabilizes the cysteine transporter xCT and promotes the synthesis of reduced glutathione (GSH) to enhance the ROS resistance of metastatic cancer cells [13]. ESRP1 expression status of melanomas played a significant role on cytolytic activity and in influencing survival of patients (ESRP1-low patients had better outcome) [16]. Jeong et al. also showed that high ESRP1 expression significantly negatively correlated with five-year survival of ovarian cancer patients [14]. Moreover, Lee et al. reported that the higher ESRP1 expression represented an unfavorable prognostic factor in prostate cancer, and increased the risk of disease progression and cancer-specific death in this disease [17].

The expression ESRP1 and its paralog ESRP2 is highly plastic, and several up-regulation and down-regulation episodes are seen in the multistep process of cancer progression, pointing out the difficulty in designing therapeutics against these molecules [18]. It is thus crucial to fully dissect the molecular events regulated by ESRP1 in CRC in order to appropriately target tumor progression. To gain further insights into the mechanism by which ESRP1 exerts a pro-tumorigenic activity in CRC cells, we performed a high throughput cDNA microarray on two cell lines, COLO320DM and HCA24, modulated for ESRP1 expression. Intriguingly, our data show, unprecedently that ESRP1 promotes the expression of and involves RAC1b in its cancer-promoting role.

## 2. Materials and Methods

### 2.1. Cell Lines and ESRP1 Expression Modulation

The CRC cell lines used in this study had been previously characterized, at both the genomic and transcriptomic level, and authenticated [19]. COLO320DM cells were cultured in RPMI (ThermoFisher Scientific, Monza, Italy), 10% FBS and penicillin/streptomycin (PS), while HCA24 and Caco-2 cells were kept in DMEM, 10% FBS and PS as previously described [12]. ESRP1 expression was modulated with shRNA cloned into pLKO.1 lentiviral vector for stable knockdown and with human ESRP1 ORF cloned into pLX304 lentiviral vector for stable overexpression [11]. RAC1b expression was transiently reduced by using siRNA against this gene by using lipofectamine as described (Table S1) [20].

### 2.2. RNA Extraction and Quantitative Real-Time PCR

RNA was extracted using the PureLink RNA kit (ThermoFisher Scientific, Monza, Italy) and cDNA prepared with the High-capacity cDNA Reverse Transcription kit (ThermoFisher Scientific, Monza, Italy). Real-time PCR (qRT-PCR) was performed to analyze target gene expression as previously described [11,21]. Gene expression was normalized to endogenous 18 s (for Taqman gene expression assays) or *GAPDH* expression. Primer sequences are reported in Table S1 [22,23,24,25,26,27,28,29,30,31].

### 2.3. cDNA Microarray Analysis 

Gene expression profiling was performed as previously described [11]. Briefly, RNA was extracted using miRNeasy Mini Kit (Qiagen, Milano, Italy), according to the manufacturer’s protocol. RNA quantification, quality assessment, cRNA synthesis, hybridization and data processing are described in the Supplementary Materials. COLO320DM cells overexpressing ESRP1 and ESRP1-silenced HCA24 cells were compared to empty and scramble controls, respectively. Bioconductor limma package was used for differential expression analysis with Benjamini–Hochberg (BH) method for False Discovery Rate (FDR) evaluation [32]. For the COLO320DM data, cut-off values were set to p-value adj < 0.05 and abs(log_2_FC) > log_2_(1.5) whereas for HCA24 *p*-value adj < 0.01 and abs(log_2_ FC) > log_2_(1.5), in order to limit the number of differentially expressed genes. Datasets have been deposited in the GEO repository (GSE180125 and GSE180126).

### 2.4. RNA-Immunoprecipitation 

RNA immunoprecipitation was performed as previously described [12]. Briefly, total cell protein extracts were obtained by incubating COLO320DM cells overexpressing ESRP1 for 5 min in cold isotonic buffer (20 mM HEPES,100 mM NaCl, 250 mM Sucrose, 5 mM MgCl_2_), a cocktail of protease inhibitors (Roche, Milan, Italy) and RNAse inhibitor (Promega, Milan, Italy) and DTT. The lysates were precleared for 1 h at 4 °C using Dynabeads protein G. Anti-ESRP1 antibody (Sigma-Aldrich, Milan, Italy) or rabbit IgG was added to the precleared lysates overnight at 4 °C and the day after, dynabeads were added for 3 h at 4 °C. After washing, the beads-bound proteins were processed for Western blot or RNA purification and qRT-PCR.

### 2.5. Protein Extraction and Western Blotting

Protein was extracted from CRC cells using RIPA buffer (50 mM Tris-HCl pH 8, 150 mM NaCl, 1% NP-40, 0.5% sodium deoxycholate, 0.1% SDS) supplemented with protease inhibitors (Complete Mini, Roche, Monza, Italia) and a cocktail of phosphatase inhibitors (5 mM sodium fluoride, 1 mM sodium vanadate and 1 mM PMSF). SDS-PAGE (Bio-rad, Segrate, Italy) was performed as previously described [12]. Antibodies used are listed in Table S2. Densitometric analysis was performed using Image Lab 6.1 software (Biorad Laboratories, Segrate, Italy).

### 2.6. Soft Agar Assay

The soft agar assay was performed as described elsewhere [33]. Briefly, 2 × 10^5^ Caco-2 cells (control and siRAC1b) were plated in the upper layer and stained after 2 months with p-nitroblue tetrazolium (NBT, Sigma, Milano, Italy) for colony visualization and quantification.

### 2.7. Enrichment Analyses

Analyses have been conducted on R free software environment for statistical computing and graphics (version 3.6.3). Bioconductor package ClusterProfiler (v3.12.0) has been used for enrichment analysis [34]. We focused on Biological Process, Cellular Component and Molecular Function of Gene Ontology (GO) resource. GO keywords with no more than 2000 associated genes were analyzed and Benjamini–Hochberg (BH) strategy for false discovery rate (FDR) was applied by using a cut-off at 0.05 both for *p*-value and *q*-value.

### 2.8. Colorectal Cancer Expression Datasets

*ESRP1*, *RAC1* and *RAC1b* expression were analyzed from CRC expression datasets as follows. RAC1 isoforms analysis was performed based on specific isoforms expression data. The data for *RAC1* (uc003spx) and *RAC1b* (uc003spw) isoforms were obtained from TCGA COAD dataset as reported at TCGA Synapse group (available online: https://www.synapse.org/#!Synapse:syn300013/tables/ (accessed on 15 July 2021) [35]. Expression data for *ESRP1* were taken from Xena Functional Genomics Explorer TCGA COAD gene expression by RNAseq (polyA + IlluminaGA) (https://xenabrowser.net/, accessed on 15 July 2021). We also performed RAC1 isoforms analysis based on percent spliced in (PSI, defined as the ratio between the number of reads supporting exon inclusion and the combined number of reads regarding inclusion and exclusion) values [36]. Ryan et al. analyzed TCGA data and showed how *RAC1b* isoform is expressed in colon tumors (COAD). We have correlated this percentage value with the ESRP1 RNAseq-HTSeq-FPKM-UQ expression value (as found in GDC TCGA Colon Cancer (COAD)) [37].

Moreover, in order to highlight possible differences in *RAC1* behavior according to *ESRP1* expression level in TCGA COAD, samples with low *ESPR1* and high *ESRP1* expression were selected. Specifically the two groups composed of samples belonging to the first and the fourth quartile, respectively, were considered.

### 2.9. Statistical Analyses

Data in bar graphs are expressed as mean ± standard deviation. Data in the whisker plot of Figure S1 are expressed as median and first and fourth quartiles. Statistical differences were determined by a 2-tailed Student’s t-test (* *p* < 0.05, ** *p* < 0.01, *** *p* < 0.001, **** *p* < 0.0001).

## 3. Results

### 3.1. Gene Expression Profiling of ESRP1-Modulated COLO320DM and HCA24 Cells 

We previously demonstrated that ESRP1 expression is absent in the highly metastatic cell line COLO320DM, while it is expressed at elevated levels in HCA24 cells [11]. These cells were chosen for the experiments as they do no bear the most common driver mutations of CRC in BRAF, KRAS, PIK3CA and NRAS [19]. COLO320 DM cells are undifferentiated tumorigenic cells that exhibit epithelial to mesenchymal transition (EMT) features as shown by the absence of epithelial markers, such as E-cadherin, EpCAM, cytokeratin, and the presence of mesenchymal markers such as vimentin. Consistently, they have strong metastatic potential in vivo as observed in experimental metastasis models [11,38,39]. On the other hand, HCA24 cells have strong epithelial features and are highly differentiated cells that generate large xenografts in vivo without any evidence of metastasis generation [40]. Thus, we re-introduced ESRP1 cDNA in COLO320DM and silenced the expression of ESRP1 in HCA24 cells. ESRP1-overexpressing COLO320DM cells showed enhanced proliferation in suspension and significantly increased the formation of macro-metastasis in the liver with respect to empty controls when delivered through the tail vein. On the other hand, ESRP1-silencing in HCA24 cells abrogated their capacity to proliferate in suspension and to form colonies in soft agar with respect to control cells [11].

**Figure 1:**
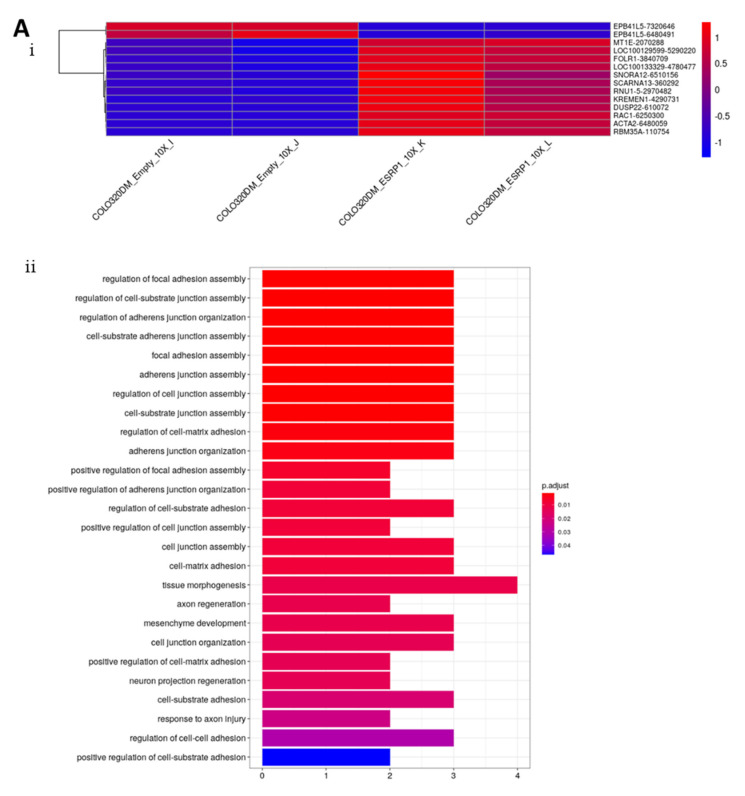

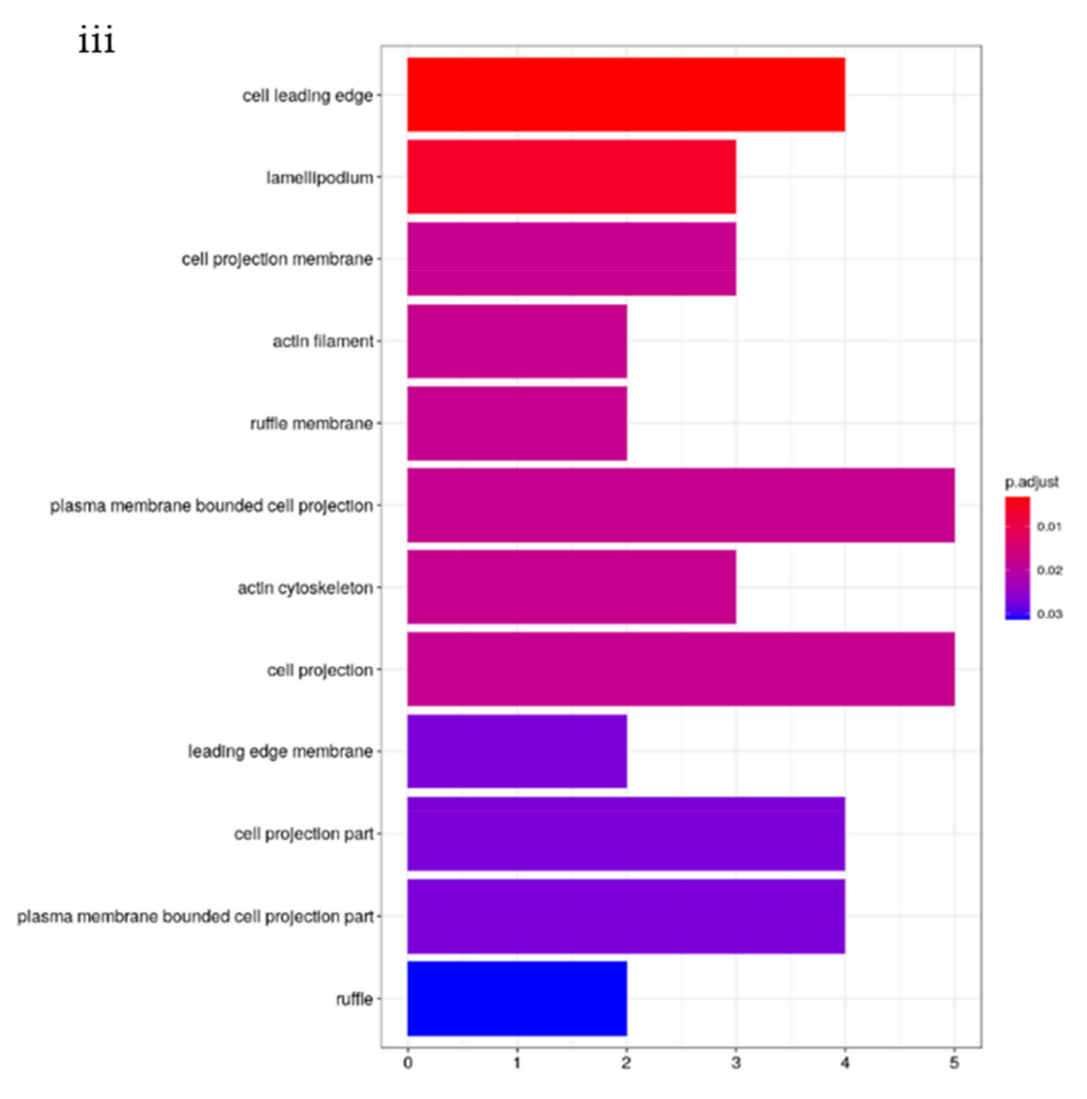

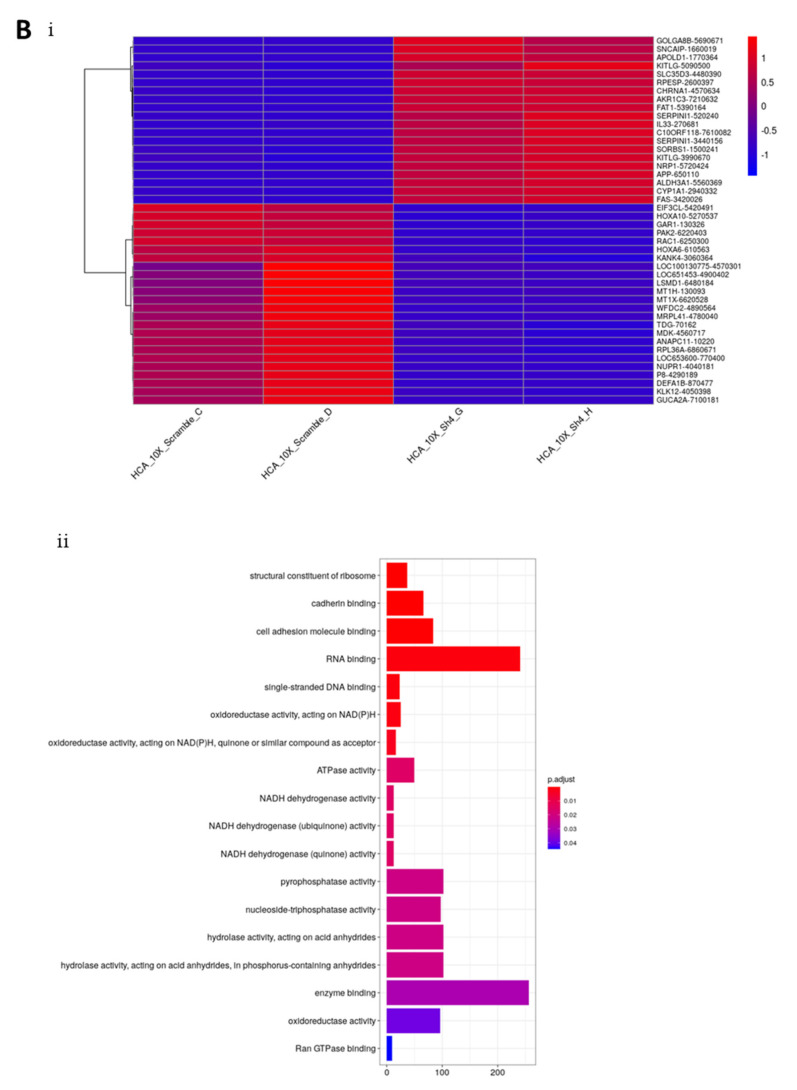

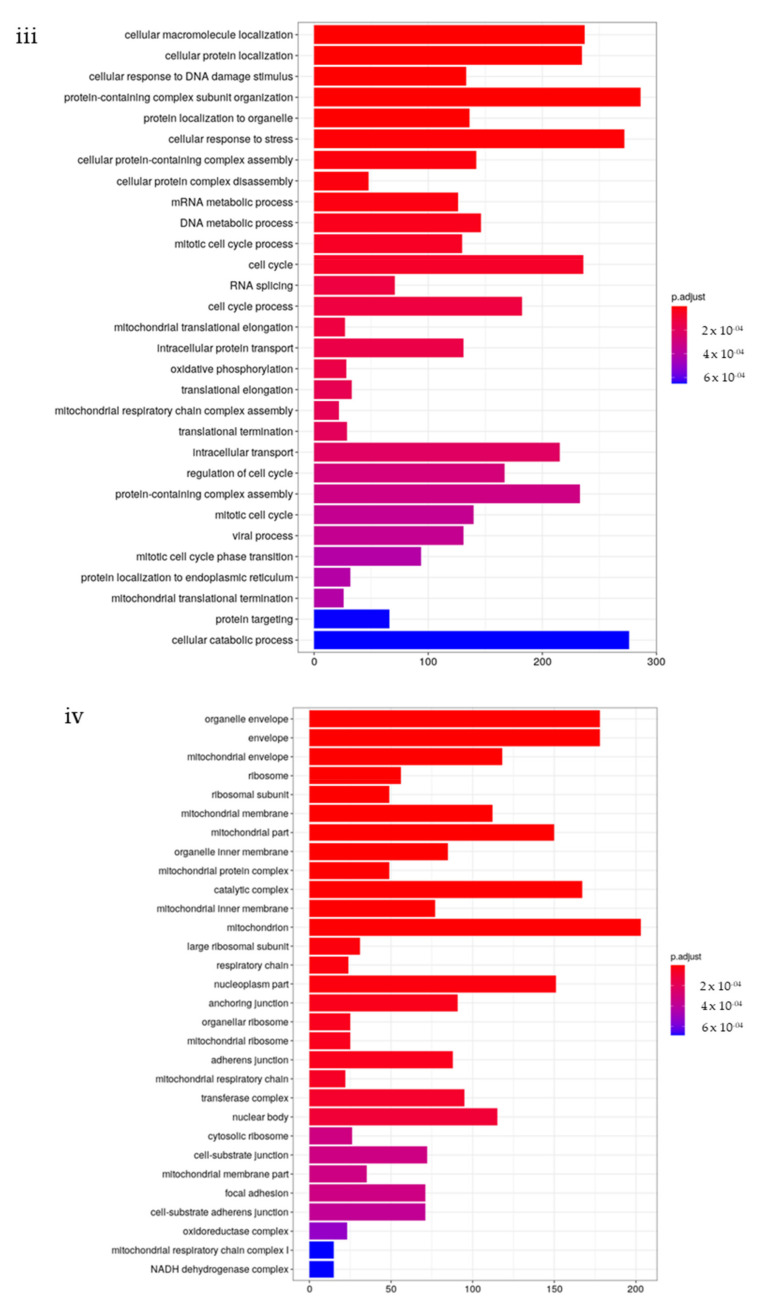
Gene expression profiling of ESRP1-modulated CRC cells. (**A**). (**i**) Heatmap of ESRP1-overexpressing and control CO-LO320DM cells. Cells are shown in columns and genes in rows (genes are reported as Gene Name and Illumina Array Address ID). Gene ontology keywords belonging to (**ii**) biological process domain and (**iii**) cellular component domain are reported on the y-axis, the number of differentially expressed genes involved in the corresponding GO term enrichment is defined on the x-axis, whereas the different colors highlight the different magnitude of the *p*-values (as in the legend). (**B**). (**i**) Heatmap of ESRP1-silenced and control HCA24 cells. Only gene showing a |log2FC| > 1.5 are considered in this graphical representation. Cells are shown in columns and genes in rows (genes are reported as Gene Name and Illumina Array Address ID). HCA24 enriched Gene Ontology keywords belonging to (**ii**) molecular function domain, (**iii**) biological process domain and (**iv**) cellular component domain are reported on the y-axis, the number of differentially expressed genes involved in the corresponding GO term enrichment is defined on the x-axis, whereas the different colors highlight the different magnitude of the *p*-values (as in the legend).

In order to investigate in more depth, the mechanism by which ESRP1 regulates anchorage-independent growth and tumorigenesis in CRC cells, we performed high throughput gene expression profiling analysis of ESRP1-modulated COLO320DM and HCA24 cells versus respective controls (Figure 1). The results show that ESRP1 overexpression in COLO320DM cells can induce changes in the expression of transcripts involved in, for example, cell–cell adhesion (biological process), and cell projection and actin cytoskeleton reorganization (cellular component) as evidenced by gene ontology analysis (Figure 1A and Table S3). Changes pertaining to cellular architecture are important drivers of tumorigenesis [41]. On the other hand, ESRP1-silencing in HCA24 cells resulted in differential expression of transcripts involved in, for instance, RNA-binding (molecular function), mRNA metabolism and translation (biological process), and ribosome (cellular component) pertaining to the role of ESRP1 in RNA biogenesis (Figure 1B and Table S4).

**Figure 2:**
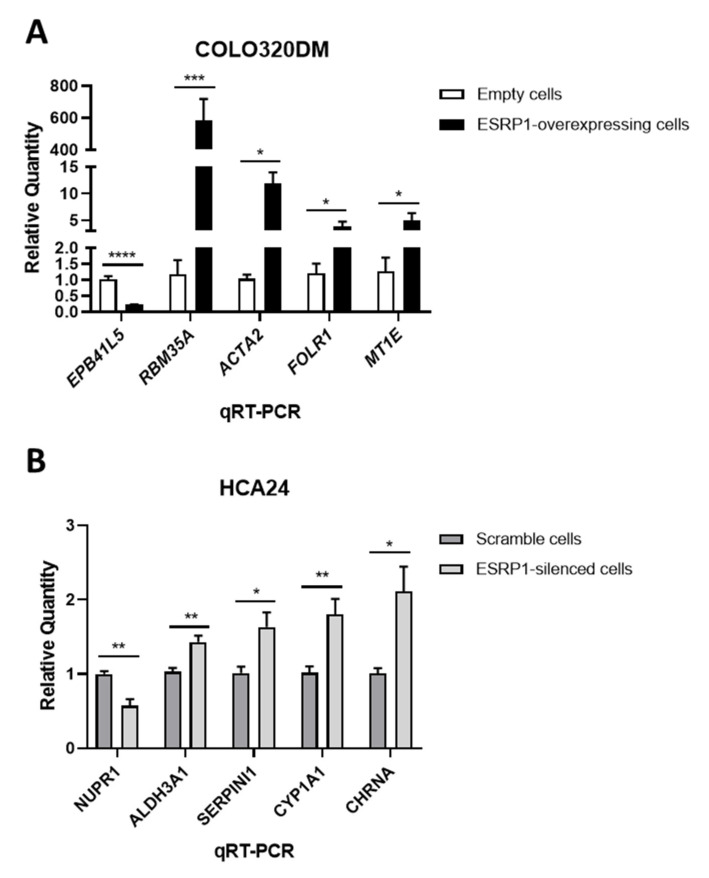
Microarray data validation. (**A**) Gene expression levels of *EPB41L5*, *RBM35A* (*ESRP1*), *ACTA2*, *FOLR1* and *MT1E* in ESRP1-modulated COLO320DM cells (*n* = 6). (**B**) Gene expression levels of *NUPR1*, *ALDH3A1*, *SERPINI1*, *CYP1A1* and *CHRNA* in ESRP1-modulated HCA24 cells (*n* = 4). Data are expressed as mean ± SEM of relative quantification using the delta–delta Ct method over control (empty and scramble in COLO320DM and HCA24 cells, respectively) cells. Normalization was made using *GAPDH* as housekeeping gene. Unpaired t-test was performed: * *p* < 0.05, ** *p* < 0.01, *** *p* < 0.001, **** *p* < 0.0001.

The cDNA microarray data were experimentally validated with qRT-PCR analysis in both cell lines. In agreement with the cDNA microarray results, *EPB41L5* expression was found to be down-regulated, while *RBM35A* (*ESRP1*), *ACTA2*, *FOLR1* and *MT1E* to be up-regulated upon ESRP1 overexpression in COLO320DM cells with respect to empty controls (Figure 2A and Table S5). On the other hand, *NUPR1* expression was found to be down-regulated, while *ALDH3A1, SERPINI1*, *CYP1A1* and *CHRNA* expression was found to be up-regulated in ESRP1-silenced HCA24 cells compared to the scramble control cells (Figure 2B and Table S6). A small number of genes were found to be commonly differentially expressed in the two cell lines upon ESRP1 modulation, as expected due to their inherent differences (Table S7). Overall, these results suggest that the modulation of ESRP1 expression levels induces gene expression changes in both CRC cell lines.

### 3.2. ESRP1 Positively Regulates RAC1b Expression in CRC Cells

**Figure 3:**
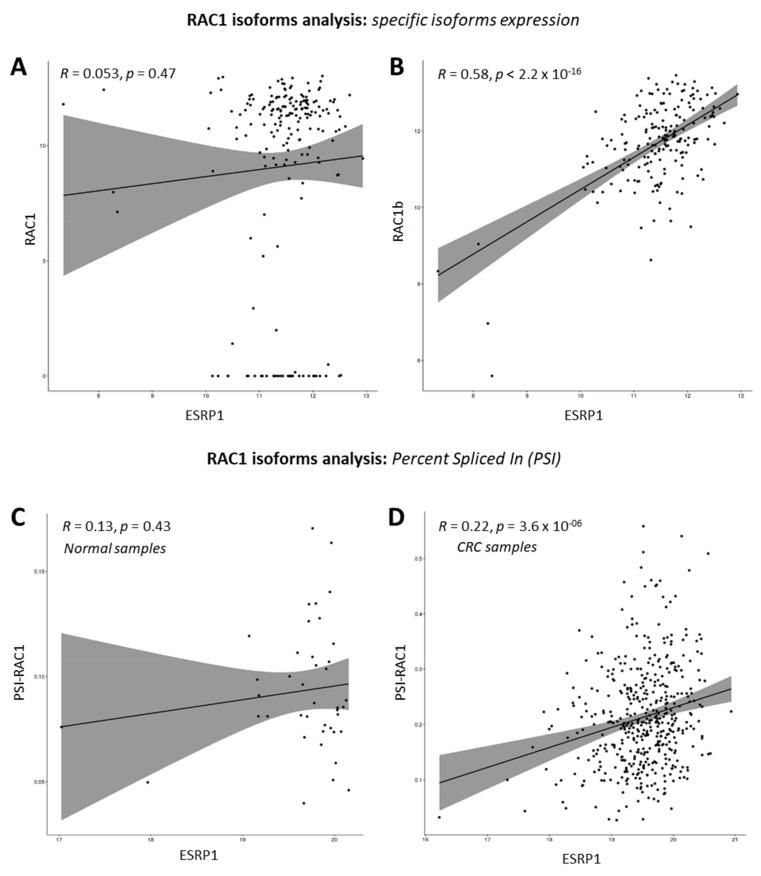
RAC1 isoforms analysis. (**A**) Pearson correlation analysis between ESRP1 and RAC1 (uc003spx) in TCGA COAD tumor samples. (**B**) Pearson correlation analysis between ESRP1 and RAC1B (uc003spw) in TCGA COAD tumor samples. This specific isoform shows a positive and statistically significant correlation. (**C**) Pearson correlation analysis between ESRP1 and PSI values related to RAC1 in TCGA COAD normal samples. (**D**) Pearson correlation analysis between ESRP1 and PSI values related to RAC1 in TCGA COAD tumor samples. In this specific tumor context, the percent spliced in values related to RAC1 shows a positive and statistically significant correlation.

Interestingly, among the most significantly differentially expressed genes (Figure 1), RAC1 topped in COLO320DM cells overexpressing ESRP1. RAC1 was also found in the list of significantly differentially expressed genes in the HCA24 cells silenced for ESRP1 expression. The probe that on the microarray platform identifies the differential expression of *RAC1* refers specifically to exon 3b, which is part of a particular isoform, *RAC1b*, of the *RAC1* gene itself (Table S8). The TCGA COAD datasets were further analyzed for correlation between *ESRP1* and *RAC1* or *RAC1b* expression. As shown in Figure 3, *RAC1b* expression, with respect to *RAC1*, showed statistically significant positive correlation with that of *ESRP1* in the CRC patient samples. A further evaluation of *RAC1b*-specific exon inclusion, using the percent spliced in (PSI) approach, showed that there is a significant and positive correlation between *ESRP1* and PSI values for *RAC1* in 452 tumor samples, but not in the corresponding 41 normal samples. There are also indications from TCGA COAD datasets, that low *ESRP1* and high *ESRP1* subsets of CRC (first and the fourth quartile) show differential expression of *RAC1* isoforms and *RAC1* PSI (Figure S1).

**Figure 4:**
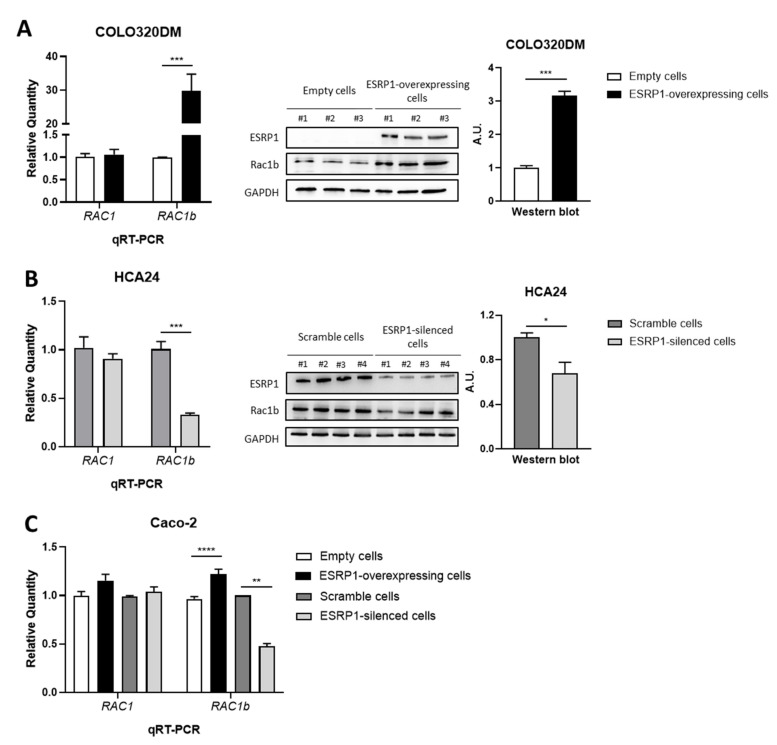
RAC1b data validation in all CRC cell lines under study. (**A**) RAC1b up-regulation in COLO320DM cells was validated by qRT-PCR (on the left) and Western blot (on the right) analysis (*n* = 5 for qRT-PCR and *n* = 3 for Western blot). (**B**) RAC1b down-regulation in HCA24 cells was validated by qRT-PCR (on the left) and Western blot (on the right) analysis (*n* = 4). (**C**) RAC1b deregulation upon ESRP1 modulation in Caco-2 cells was validated by qRT-PCR (*n* = 3). *RAC1* expression levels were evaluated by qRT-PCR analysis in all CRC cell lines. Data are expressed as mean ± SEM of relative quantification using the delta–delta Ct method over control (empty and scramble) cells. Normalization was made using *GAPDH* as housekeeping gene. Unpaired t-test was performed: * *p* < 0.05, ** *p* < 0.01, *** *p* < 0.001, **** *p* < 0.0001.

We thus focused our attention on RAC1b. RAC1b and ESRP1 showed a similar expression behavior according to the differential expression analysis in both CRC cell lines (Figure 4A,B). CDNA microarray data were validated both at the RNA and protein levels by qRT-PCR and Western blot analyses, respectively, in independent experiments. Consistently, RAC1b was found to be up-regulated and down-regulated in COLO320DM ESRP1-overexpressing and HCA24 ESRP1-silenced cells, respectively, compared to their respective controls (Figure 4A,B). On the other hand, RAC1 mRNA expression level was not significantly different among the ESRP1-modulated CRC cells (Figure 4A,B).

In order to analyze if ESRP1 and RAC1b also showed a similar trend in expression in a normal colon-like cell line, we analyzed RAC1b expression in Caco-2 cells, which express intermediate levels of ESRP1 (comparable to the normal colon) and which underwent both ESRP1 overexpression and silencing as previously reported [11]. Interestingly, *RAC1b* expression increased when ESRP1 was over-expressed in Caco-2 cells, while its expression decreased when ESRP1 expression was silenced (Figure 4C). Of note, *RAC1* expression was not significantly different among the ESRP1-modulated Caco-2 cells (Figure 4C).

Overall, these results suggest that RAC1b expression, both at protein and mRNA levels, is positively regulated by ESRP1 in all CRC cells under study. On the contrary, total RAC1 mRNA level did not change, thus allowing us to speculate that ESRP1 can directly lead to a switch from the canonical to the alternatively spliced variant RAC1b.

### 3.3. The Expression of Known Regulators of Rac1b Splicing Is Not Affected by ESRP1 Modulation in CRC Cells

Previous studies have reported ASF/SF2 (also known as SFRS1) and SRp20 (also known as SFRS3) as the master splicing regulators of RAC1 [25]. Moreover, it has also been shown that SRPK1 and GSK3β are involved in the regulation of such splicing factors [42]. In order to investigate whether, in our cellular models, these players can somehow be involved in orchestrating RAC1 splicing upon ESRP1 modulation, we interrogated our cDNA microarray data for SFRS1, SFRS3, SRPK1 and GSK3β expression. Of note, there was no statistically significant differences in the datasets analyzed, thus suggesting that other mechanisms might be involved (Table 1).

### 3.4. RAC1 mRNA Co-Immunoprecipitates with ESRP1

ESRP1 is a well-known splicing factor capable of binding the GU-rich sequence motifs of several mRNAs [43]. Of note, a GU-rich ESRP-binding consensus sequence (UGGUGGGUG) was found 274 bp upstream of exon 3b [18]. Based on our cDNA microarray data, as well as Western blot and qRT-PCR analyses, we speculated whether ESRP1 can directly bind pre-mRNA *RAC1*, thereby promoting the inclusion of exon 3b and generating RAC1b isoform at the expense of the canonical one.

**Figure 5:**
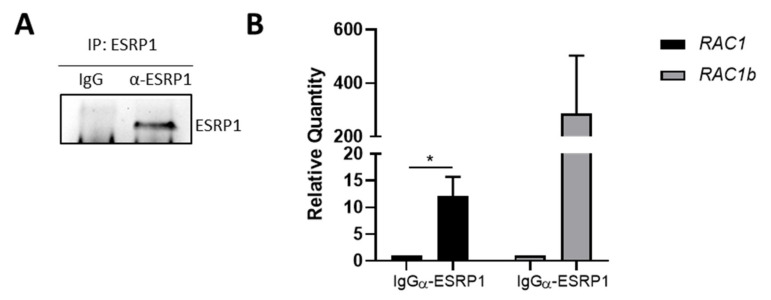
RNA-immunoprecipitation (RNA-IP) in ERSP1-overexpressing COLO320DM CRC cells. (**A**) Western blot analysis of ESRP1 immunoprecipitation. Rabbit IgG was used as negative control. (**B**) qRT-PCR analysis of ESRP1- and IgG-bound transcripts. Gene expression levels of *RAC1* and *RAC1b* in three independent experiments of RNA-IP. Data are expressed as mean ± SEM of quantification relative to IgG negative control. Unpaired t-test was performed: * *p* < 0.05.

To this end, we performed RNA-immunoprecipitation with anti-ESRP1 antibody on ESRP1-overexpressing COLO320DM cells, in which *RAC1b* was one of the most significantly differentially expressed genes, with respect to empty controls, highlighted by the cDNA microarray data (Figure 1). IgG was used as negative control. Interestingly, qRT-PCR analysis of ESRP1-bound transcripts revealed the presence of both *RAC1* and *RAC1b* isoform compared to IgG controls (Figure 5). These results suggest that ESRP1 may have a direct role in RAC1 alternative splicing and on RAC1b expression.

### 3.5. RAC1b Silencing Affects Anchorage-Independent Tumor Growth in CRC Cells

**Figure 6:**
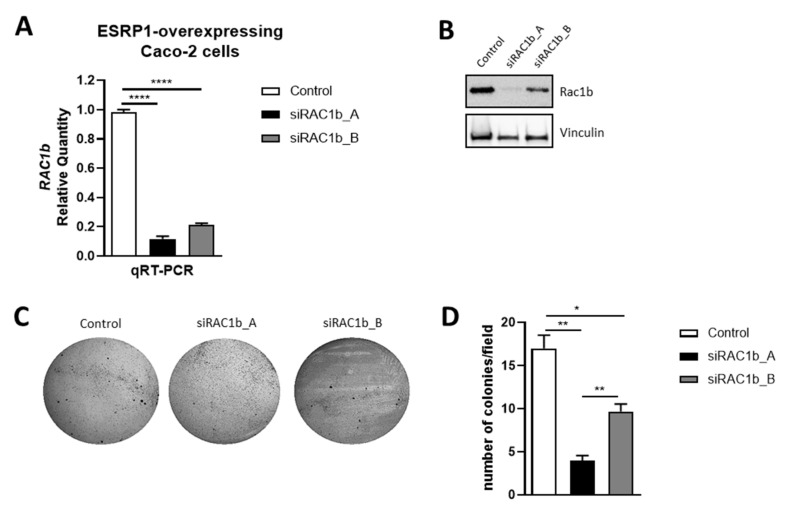
RAC1b silencing in ESRP1-overexpressing Caco-2 cells. (**A**) Gene expression levels of *RAC1b* in Caco-2 cells (*n* = 3). Data are expressed as mean ± SEM of relative quantification using the delta–delta Ct method over IgG negative control, and normalized using *GAPDH* as housekeeping gene. Unpaired t-test was performed: ****p < 0.0001. (**B**) Western blot analysis of RAC1b in Caco-2 cells. (**C**–**D**). Soft agar assay. (**C**) Representative images and (**D**) quantification of colonies (*n* = 3). Data are expressed as mean ± SEM. Unpaired t-test was performed: * *p* < 0.05, ** *p* < 0.01.

We have previously shown that ESRP1-overexpressing Caco-2 cells are capable of generating colonies in soft agar with respect to empty controls [11]. Caco-2 overexpressing cells recapitulated some of the early oncogenic changes, such as enhanced proliferation and growth in anchorage-independency. In order to investigate whether ESRP1 exerts its pro-tumorigenic function through RAC1b, the ESRP1-overexpressing Caco-2 cells were transfected with two siRNAs against RAC1b. Cells were analyzed after 48 h transfection for the expression of RAC1b. Both siRNAs efficiently down-regulated RAC1b mRNA and protein expression in these cells (Figure 6A,B). Interestingly, RAC1b silencing reduced the number of ESRP1-overexpressing Caco-2 cells colonies in soft agar (Figure 6C,D). Overall, these results suggest that ESRP1, by directly regulating the splicing of RAC1 and the expression of RAC1b, is capable of regulating the anchorage-independent growth and tumorigenesis in CRC cells.

## 4. Discussion

It has become increasingly clear that posttranscriptional gene regulation strictly regulates every cancer hallmark [44,45,46,47]. Indeed, RBPs, by orchestrating mRNA processing and translation, are thought to play a crucial role in tumor development. As an RBP, ESRP1 has been shown to be involved in several cellular processes, such as alternative splicing and regulation of translation, as well as mRNA stability [21,48]. We previously demonstrated that in some CRC cases, ESRP1 is aberrantly overexpressed, and acts as an oncogene [11,12]. In the present study, we further investigated the underlying molecular mechanisms by which ESRP1 exerts its pro-tumorigenic function by performing large-scale gene expression profiling on two different CRC cell lines, in which ESRP1 expression was modulated. Intriguingly, comparison of datasets of our cDNA microarray data revealed that few genes were commonly differentially expressed between the two cells lines (Table S7). This was expected due to the different oncogenic pathways activated in the strongly mesenchymal COLO320DM cells, in which ESRP1 was re-expressed, and the epithelial HCA24 cells, in which ESRP1 expression was silenced. *RAC1b* was one of the most significantly differentially expressed gene present in both CRC cell lines (i.e., HCA24 and COLO320DM), and was hence selected for further experiments. ESRP1 and RAC1b expression showed similar trend. These results were further confirmed in a third normal colon-like cell line (Caco-2) which was modulated for ESRP1 expression. We provide herein the first evidence that ESRP1 positively regulates the expression level of the alternatively spliced RAC1b isoform. Of note, bioinformatic analysis of TGCA COAD dataset further corroborated our experimental results. No changes in the relative expression of *RAC1* mRNA were observed upon ESRP1 modulation. We thus postulate that ESRP1 is capable of regulating *RAC1* pre-mRNA splicing, thereby promoting the inclusion of variant exon 3b and the generation of RAC1b isoform. An RNA-based regulation of RAC1b may also be possible [49]. Silencing of RAC1b in ESRP1-overexpressing Caco-2 cells partially reverted the oncogenic phenotype of these cells (soft agar assay), thus supporting the fact that ESRP1 exerts its pro-tumorigenic function by acting through RAC1b.

Mammalian *RAC1* gene can give rise to two alternative transcripts, the predominant *RAC1* and the alternative isoform *RAC1b*, which contains an additional 57 nucleotides-long exon 3b. Importantly, *RAC1b* has been found aberrantly expressed in a subset of CRC tumors [20]. The in-frame insertion of 19 amino acid results in the constitutive activation of this small GTPase [50,51,52]. Over the years, RAC1b has been recognized as a pro-tumorigenic player, due to its involvement in several key processes including cell cycle progression and apoptosis resistance [53]. Moreover, RAC1b plays a critical role in the development of resistance to cancer therapies. RAC1b may be considered as a predictor of chemotherapy efficacy in metastatic CRC [54,55]. Indeed, Alonso-Espinaco et al. showed that RAC1b is a poor survival marker in KRAS/BRAF wild-type CRC patients treated with first-line FOLFOX/XELOX therapy [54]. Likewise, Goka et al. later demonstrated that CRC cells (i.e., HT116 and HT29) up-regulated RAC1b expression upon chemotherapy treatment, thereby promoting NF-κB pathway and cell survival gene transcription [55]. The inhibition of RAC1 prevents the activation of these pathways associated to chemotherapy treatment and increases the sensitivity of the cells to oxaliplatin and 5-FU.

Two independent studies have demonstrated so far that ESRP1 negatively regulates RAC1b [18,56]. In particular, Ishii et al. showed that the down-modulation of ESRP1 in human head and neck squamous cell carcinoma cell lines (i.e., SAS and HSC4) led to the induction of RAC1b expression [18]. Moreover, Deng et al., demonstrated that the overexpression of ESRP1 inhibited the generation of RAC1b in the ovarian cancer cell line SKOV3 [56]. However, no direct interaction between ESRP1 and *RAC1b* was shown in these studies. On the other hand, our results clearly show that in three different CRC cell lines, ESRP1 expression correlates with that of RAC1b and that the ESRP1-containing ribonuclear protein complex can bind both *RAC1* and *RAC1b* mRNA. Importantly, each alternative splicing event is controlled by multiple RBPs, rather than just a single RBP [57]. Moreover, each cell type expresses a distinct array of RBPs, thus dictating a distinct pattern of alternatively splice products. Thus, the cellular context matters and each cell distinguishes itself by the relative abundance of each RBP, which becomes the major determinant of pre-mRNA splicing. Thus, future studies should focus on analyzing the ESRP1-containing complexes to determine which proteins and RNA species are present under a given condition.

The RNA-binding protein ESRP1 is as a key splicing regulator related to EMT [58]. In particular, ESRP1 directs an epithelial splicing program. Specifically, it binds and regulates the splicing of several EMT-related mRNA targets (i.e., *FGFR2*, *CD44*, *ENAH* and *CTNND1*), and orchestrates the switch to the epithelial-specific transcript variants at the expense of the mesenchymal ones [48,59]. Moreover, it has also been demonstrated that EMT-associated transcription factors (i.e., δEF1, SIP1, SNAIL, SLUG and TWIST) down-regulate ESRP1 [60,61,62,63]. Recently, the alternative splice variant RAC1b has been recognized as the “guardian of the epithelial phenotype”, due to its ability to interfere with the TGF-β-induced cell migration and EMT [64]. Taken together, it may be possible that ESRP1 and RAC1b work in concert to ensure the maintenance of an epithelial phenotype, as pointed out by the analysis of TCGA COAD data on *ESRP1* or *RAC1b* versus representative EMT genes (E-cadherin and Vimentin), as well as to modulate chemoresistance development (Figure S2) [55].

## 5. Conclusions

To our knowledge, this is the first report of ESRP1 exerting its pro-tumorigenic function through RAC1b. ESRP1 is capable of binding *RAC1* mRNA, thereby promoting the inclusion of the variant exon 3b and modulating RAC1b expression. Our data solicits further work to address more in depth the molecular mechanisms of ESRP1-induced regulation of RAC1b expression and its biological effect in CRC progression for instance by employing genetically modified mice models.

## Figures and Tables

**Table 1:** Expression of splicing factors to be directly involved in RAC1b splicing. cDNA microarray data generated on the two ESRP1-modulated cell lines, COLO320DM and HCA24 were analyzed for expression of RAC1b. COLO320 DM, logFC = 1.45, *p*-Value Adjusted = 0.003; HCA24, logFC = −1.88, *p*-Value Adjusted = 2.77 × 10^-05^.

CRC Cell Line	Gene	logFC	*p*-Value	*p*-ValueAdj
COLO320DM (vs. empty cells)	*SFRS1*	−0.489781297	0.012866813	0.365144154
	*SRPK1*	−0.357614922	0.025871261	0.42769659
	*SFRS3*	−0.450996868	0.030901422	0.446890088
	*SFRS3*	−0.161124083	0.293793057	0.791369823
	*GSK3β*	−0.163911091	0.288342371	0.788788089
HCA24 (vs. scramble cells)	*SFRS1*	0.173660122	0.229342737	0.587853808
	*SRPK1*	0.070938569	0.581454363	0.844252266
	*SFRS3*	0.377887018	0.062396779	0.314723978
	*SFRS3*	−0.034194091	0.847903379	0.95510413
	*GSK3β*	−0.466411002	0.002329757	0.054686882

## Data Availability

Datasets have been deposited in GEO database, with accession numbers GSE180125 and GSE180126.

## Supplementary Materials

The following are available online at https://www.mdpi.com/article/10.3390/cancers13164092/s1, Figure S1: Differential expression analysis of RAC1 in TCGA COAD samples, as reported in the Material and Methods section, Figure S2: Pearson correlation coefficient and statistical analysis between representative EMT genes and ESRP1 and RAC1b, respectively, Table S1: Primers and SiRNA sequences used in the present study; Table S2: Antibodies used in the present study; Table S3: Gene ontology analysis of genes differentially expressed in the COLO320DM cells, Table S4: Gene ontology analysis of genes differentially expressed in the HCA24 cells., Table S5: List of differentially expressed genes in the COLO320DM cells, Table S6: List of differentially expressed genes in the HCA24 cells; Table S7: Table with the common genes found differentially expressed in COLO320DM and HCA24 cell lines; Table S8: RAC1 probe information on Illumina cDNA microarray platform.
