# Supplementary material for: The RNA-Binding Protein ESRP1 Modulates the Expression of RAC1b in Colorectal Cancer Cells
Source: Cancers (Basel). 2021 Aug 13;13(16):4092. doi: 10.3390/cancers13164092 (PMC8392041; doi:10.3390/cancers13164092)
Supplement: Supplementary file 1 [file cancers-13-04092-s001.zip › cancers-1297407-supplementary.pdf]

cancers

MDPI

Supplementary Materials: The RNA-Binding Protein ESRP1 Modulates the Expression of RAC1b in Colorectal Cancer Cells

Marta Manco, Ugo Ala, Daniela Cantarella, Emanuela Tolosano, Enzo Medico [3], Fiorella Altruda and Sharmila Fagoonee

cDNA microarray analysis

The quantification and quality analysis of RNA was performed on a Bioanalyzer 2100 (Agilent, Cernusco sul Naviglio, Milano, Italy), using RNA 6000 nano Kit (Agilent, Cernusco sul Naviglio, Milano, Italy). Synthesis of cDNA and biotinylated cRNAwas performed using the IlluminaTotalPrep RNA Amplification Kit (ThermoFisher Scientific, Monza, Italy), according to the manufacturer's protocol using 500 ng of total RNA. Quality assessment and quantification of cRNAswere performed with Agilent RNA kits on Bioanalyzer 2100. Hybridization of cRNAs (750 ng) was carried out using Illumina Human 48 k gene chips (Human HT-12 V4 BeadChip) (Illumina, Milan, Italy) . Array washing was performed using Illumina High Temp Wash Buffer for 10 min at 55 °C, followed by staining using streptavidin- Cy3 dyes (Amersham Biosciences, Amersham, UK). Probe intensity data were obtained and normalized (by Cubic Spline normalization) using the Illumina Genome Studio software (Genome Studio V2011.1) and subsequent data processing included log2 transformation and selection of genes differentially expressed [1]. Isoform expression values, x, have been transformed into log2(x+1) to make them consistent with the data provided by other TCGA databases.

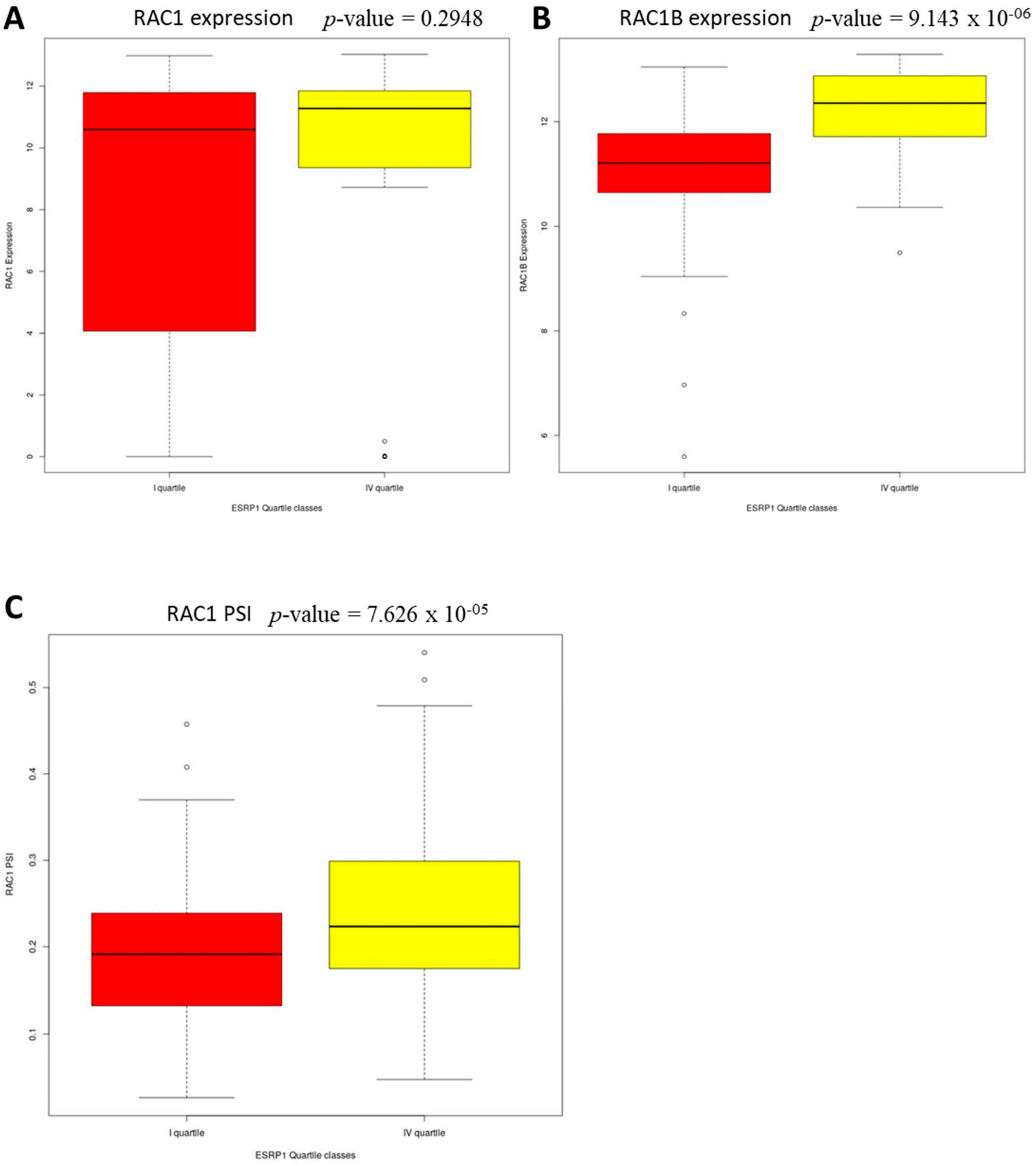

Figure S1. Differential expression analysis of RAC1 in TCGA COAD samples, as reported in the Material and Methods section. Unpaired Student *t*-test has been used to assess statistical significance. Samples have been subdivided into two classes according to ESRP1 expression values and, specifically, those belonging to the first and the fourth quartiles are used for the comparison of RAC1 expression: (**A**) Pearson correlation analysis between ESRP1 and RAC1 (uc003spx) in TCGA COAD tumor samples. RAC1 does not show a statistically significant differential expression in the samples with low and high ESRP1 values; (**B**) Pearson correlation analysis between ESRP1 and RAC1B (uc003spw) in TCGA COAD tumor samples. RAC1b shows a statistically significant differential expression in the samples with low and high ESRP1 values; (**C**) Pearson correlation analysis between ESRP1 and PSI values related to RAC1 in TCGA COAD Tumor Samples. RAC1_PSI values show a statistically significant difference in the samples with low and high ESRP1 values.

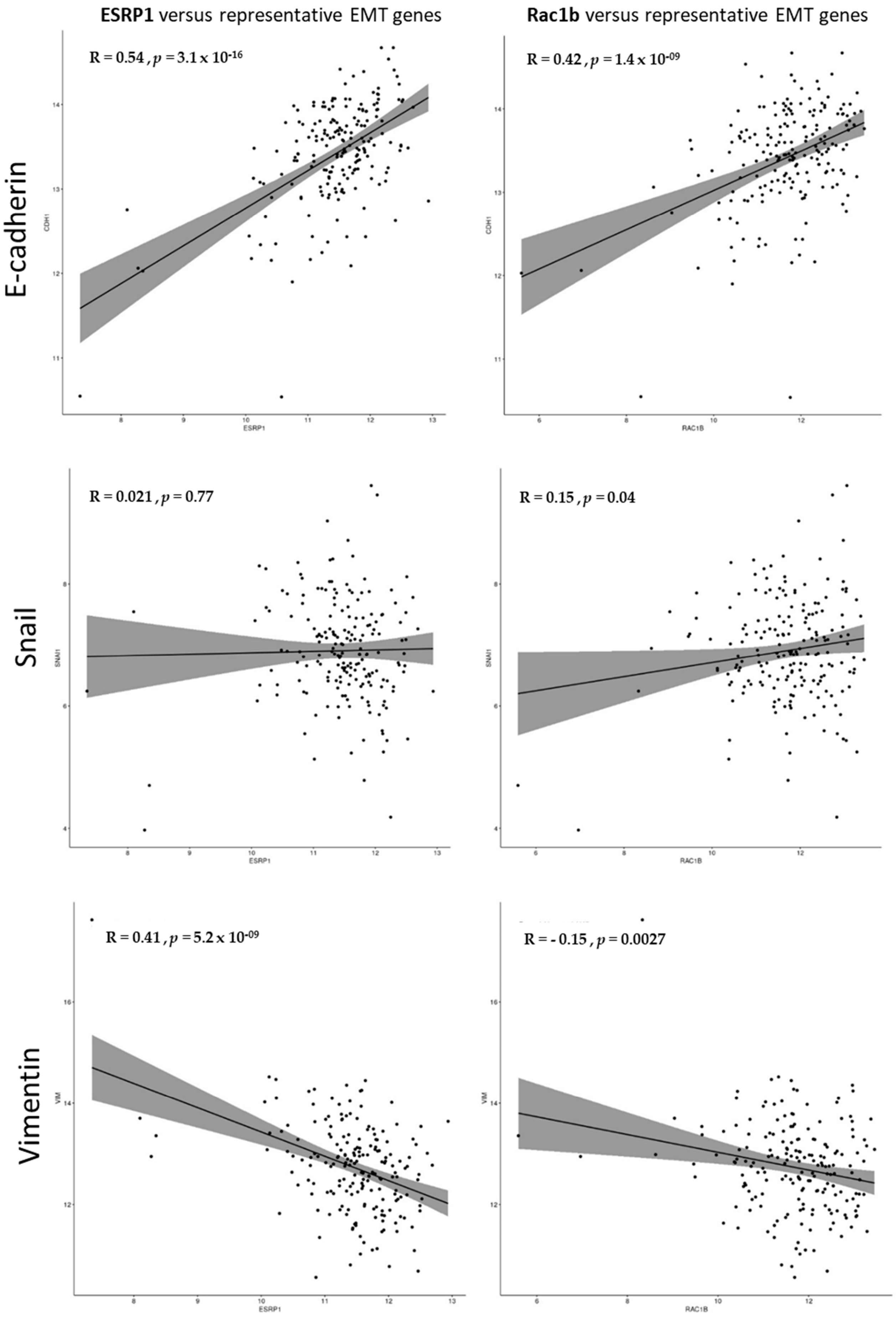

ESRP1 versus representative EMT genes
Rac1b versus representative EMT genes
E-cadherin
R = 0.54 , p = 3.1 x 10^{-16}
R = 0.42 , p = 1.4 x 10^{-09}
CDH1
ESRP1
RAC1B
Snail
R = 0.021 , p = 0.77
R = 0.15 , p = 0.04
SNAI1
ESRP1
RAC1B
Vimentin
R = 0.41 , p = 5.2 x 10^{-09}
R = - 0.15 , p = 0.0027
VIM
ESRP1
RAC1B

Figure S2. Pearson correlation coefficient and statistical analysis between representative EMT genes and ESRP1 and RAC1b, respectively. The expression data for *RAC1b* (uc003spw) isoforms were obtained from TCGA COAD dataset as reported in Colorectal cancer expression datasets in the Material and Methods section of the main text and Text S1. Expression data for *ESRP1* were taken from Xena Functional Genomics Explorer TCGA COAD gene expression by RNAseq (polyA + IlluminaGA). Specifically, E-Cadherin shows a significant positive correlation with both ESRP1 and RAC1b, Vimentin a significant negative correlation with both ESRP1 and RAC1b whereas Snail is characterized by a non-significant correlation with ESRP1 and by a slight significant positive correlation with RAC1b.

Table S1. qRT-PCR primers and siRNAs used in this study.

**Primers**	**5′-3′ sequence**<br>**Forward**<br>**Reverse**	**SYBR Green or Roche UPL Probe**	**Reference**
ESRP1 (RBM35A)	CCCACCGCCATGTAAGTT<br>GCAGGAGCTGGAAATGTGTAG	UPL#52	
ACTA2	CTATGAGGGCTATGCCTTGCC<br>GCTCAGCAGTAGTAACGAAGGA	SYBR green	[2]
EPB41L5	GCACCTCTTCTTCTCTTA<br>ACTCCTCTCAATGACAAG	SYBR green	-
ALDH3A1	GCAGACCTGCACAAGAATGA<br>TGTAGAGCTCGTCCTGCTGA	SYBR green	[3]
CYP1A1	CACCTCCAAGATCCCTACACTGA<br>ACCAGACAGAAGATGACAGAGGC	SYBR green	[4]
RAC1b	GGGCAAAGACAAGCCGATTG<br>CGGACATTTTCAAATGATGCAGG	SYBR green	[5]
RAC1	CTGATGCAGGCCATCAAGT<br>CAGGAAATGCATTGGTTGTTC	SYBR green	[5]
GAPDH	ACAACTTTGGTATCGTGGAAGG<br>GCCATCACGCCACAGTTTC	SYBR green	[6]
FOLR1	GGCATTTCATCCAGGACACC<br>CTTCCCACCATTGCTCACAG	SYBR green	[7]
NUPR1	GGAAAGGTCGCACCAAGAGAG<br>ACCAGTTTCCTCTCGTGCCC	SYBR green	[8]
SERPINI1	TAGCCGTGGCCAACTACATC<br>GGCAGCATCAAAATCCCTTG	SYBR green	[9]
CHRNA1	GCTCTGTCGTGGCCATCAA<br>CACTCCCCGCTCTCCATG	SYBR green	[10]
MT1E	GGGCTCCATTCTGCTTTCCA<br>TTGGGGTCCATTTCGAGCAA		[11]
**siRNA**	**5′-3′ sequence**	SYBR green	**Reference**
siRAC1b_A	CAG UUG GAG AAA CGU ACG GTT		[12]
siRAC1b_B	CGU ACG GUA AGG AUA UAA CTT		[12]
siControl	GGC UAC GUC CAG GAG CGC ACC TT		[12]

Table S2. Antibodies used in this study.

**Antibody**	**Species**	**Company**
ESRP1 (RIP)	Rabbit	Sigma-Aldrich, Milan, Italy, cat. no. HPA023719
ESRP1 (Western Blot)	Rabbit	Thermo Fisher Scientific, Monza, Italy, cat. no. PA5-21109
RAC1b	Rabbit	EMD Millipore, Milan, Italy, cat. no. 09-271
GAPDH	Rabbit	EMD Millipore, Milan, Italy, cat. no. MAB374
Vinculin	Mouse	In-house

Supplementary legends:

Table S3. Gene Ontology analysis of genes differentially expressed in the COLO320DM cells. The COLO320 DM enriched Gene Ontology keywords belonging to biological process (BP) and cellular component (CC) domains are reported. The different fields highlight the GO ID, its description, the nominal *p*-value, the corresponding adjusted p-value (p.adjust) and *q*-value, and finally the list of genes (gene ID) that are involved in the enrichment.

Table S4. Gene Ontology analysis of genes differentially expressed in the HCA24 cells. The HCA24 enriched Gene Ontology keywords belonging to molecular function (MF), biological process (BP) and cellular component (CC) domains are reported. The different fields highlight the GO ID, its description, the nominal *p*-value, the corresponding adjusted *p*-value (p.adjust) and *q*-value, and finally the list of genes (gene ID) that are involved in the enrichment.

Table S5. List of differentially expressed genes in the COLO320DM cells. Genes are identified by the Array Address ID (Probe), Transcript (Transcr), Gene Name (Gene), the log Fold Change (logFC), the nominal p-Value (pVal) and the adjusted p-Value (pValAdj) as in the Material and Methods and Text S1. Bioconductor limma package was used for differential expression analysis with Benjamini–Hochberg (BH) method for False Discovery Rate (FDR) evaluation. Cut-off values were set to p-value adj < 0.05 and abs(log_2FC) > log_2(1.5).

Table S6. List of differentially expressed genes in the HCA24 cells. Genes are identified by the Array Address ID (Probe), Transcript (Transcr), Gene Name (Gene), the log Fold Change (logFC), the nominal p-Value (pVal) and the adjusted p-Value (pValAdj) as in the Material and Methods and Text S1. Bioconductor limma package was used for differential expression analysis with Benjamini–Hochberg (BH) method for False Discovery Rate (FDR) evaluation. Cut-off values were set to *p*-value adj < 0.01 and abs(log_2 FC) > log_2(1.5).

Table S7. Table with the common genes found differentially expressed in COLO320DM and HCA24 cell lines. Gene symbol, Illumina transcript ID and Illumina probe ID are associated to the corresponding log Fold Change values, their nominal *p*-values and Adjusted *p*-values.

Table S8. RAC1 probe information on Illumina cDNA microarray platform.

Reference

1. Warzecha, C.; Sato, T.K.; Nabet, B.; Hogenesch, J.B.; Carstens, R.P. ESRP1 and ESRP2 Are Epithelial Cell-Type-Specific Regulators of FGFR2 Splicing. *Mol. Cell* **2009**, *33*, 591–601, doi:10.1016/j.molcel.2009.01.025.
2. Wang, X.; Jin, H.; Jiang, S.; Xu, Y. MicroRNA-495 inhibits the high glucose-induced inflammation, differentiation and extracellular matrix accumulation of cardiac fibroblasts through downregulation of NOD1. *Cell. Mol. Biol. Lett.* **2018**, *23*, 1–13, doi:10.1186/s11658-018-0089-x.
3. Duong, H.-Q.; You, K.S.; Oh, S.; Kwak, S.-J.; Seong, Y.-S. Silencing of NRF2 Reduces the Expression of ALDH1A1 and ALDH3A1 and Sensitizes to 5-FU in Pancreatic Cancer Cells. *Antioxidants* **2017**, *6*, 52, doi:10.3390/antiox6030052.
4. Divi, R.L.; Lindeman, T.L.E.; Shockley, M.E.; Keshava, C.; Weston, A.; Poirier, M.C. Correlation between CYP1A1 transcript, protein level, enzyme activity and DNA adduct formation in normal human mammary epithelial cell strains exposed to benzo[a]pyrene. *Mutagen.* **2014**, *29*, 409–417, doi:10.1093/mutage/geu049.
5. Gonçalves, V.; Matos, P.; Jordan, P. Antagonistic SR proteins regulate alternative splicing of tumor-related Rac1b downstream of the PI3-kinase and Wnt pathways. *Hum. Mol. Genet.* **2009**, *18*, 3696–3707, doi:10.1093/hmg/ddp317.
6. Xu, X.; Li, S.; Lin, Y.; Chen, H.; Hu, Z.; Mao, Y.; Xu, X.; Wu, J.; Zhu, Y.; Zheng, X.; et al. MicroRNA-124-3p inhibits cell migration and invasion in bladder cancer cells by targeting ROCK1. *J. Transl. Med.* **2013**, *11*, 276–276, doi:10.1186/1479-5876-11-276.
7. Jia, L.; Li, J.; Li, P.; Liu, D.; Li, J.; Shen, J.; Zhu, B.; Ma, C.; Zhao, T.; Lan, R.; et al. Site-specific glycoproteomic analysis revealing increased core-fucosylation on FOLR1 enhances folate uptake capacity of HCC cells to promote EMT. *Theranostics* **2021**, *11*, 6905–6921, doi:10.7150/thno.56882.
8. Yu, J.; Zhu, H.; Li, R.; Jiang, Q.; Luan, W.; Shi, J.; Liu, P. Oncogenic Role of NUPR1 in Ovarian Cancer. *OncoTargets Ther.* **2020**, *ume 13*, 12289–12300, doi:10.2147/ott.s262224.
9. Matsuda, Y.; Miura, K.; Yamane, J.; Shima, H.; Fujibuchi, W.; Ishida, K.; Fujishima, F.; Ohnuma, S.; Sasaki, H.; Nagao, M.; et al. SERPINI1 regulates epithelial–mesenchymal transition in an orthotopic implantation model of colorectal cancer. *Cancer Sci.* **2016**, *107*, 619–628, doi:10.1111/cas.12909.
10. Lam, D.C.-L.; Girard, L.; Ramirez, R.D.; Chau, W.-S.; Suen, W.-S.; Sheridan, S.; Tin, V.P.; Chung, L.-P.; Wong, M.P.; Shay, J.W.; et al. Expression of Nicotinic Acetylcholine Receptor Subunit Genes in Non–Small-Cell Lung Cancer Reveals Differences between Smokers and Nonsmokers. *Cancer Res.* **2007**, *67*, 4638–4647, doi:10.1158/0008-5472.can-06-4628.
11. Masiulionytė, B.; Valiulytė, I.; Tamašauskas, A.; Skiriutė, D. Metallothionein Genes are Highly Expressed in Malignant Astrocytomas and Associated with Patient Survival. *Sci. Rep.* **2019**, *9*, 5406, doi:10.1038/s41598-019-41974-9.

12. Matos, P.; Oliveira, C.; Velho, S.; Gonçalves, V.; Da Costa, L.T.; Moyer, M.P.; Seruca, R.; Jordan, P. B-RafV600E Cooperates With Alternative Spliced Rac1b to Sustain Colorectal Cancer Cell Survival. *Gastroenterol.* **2008**, *135*, 899–906, doi:10.1053/j.gastro.2008.05.052.
