# Supplementary material for: The RNA-Binding Protein ESRP1 Modulates the Expression of RAC1b in Colorectal Cancer Cells
Source: Cancers (Basel). 2021 Aug 13;13(16):4092. doi: 10.3390/cancers13164092 (PMC8392041; doi:10.3390/cancers13164092)
Supplement: Supplementary file 1 [file cancers-13-04092-s001.zip › Uncropped blots_Manco et al.pptx]

## Slide 1
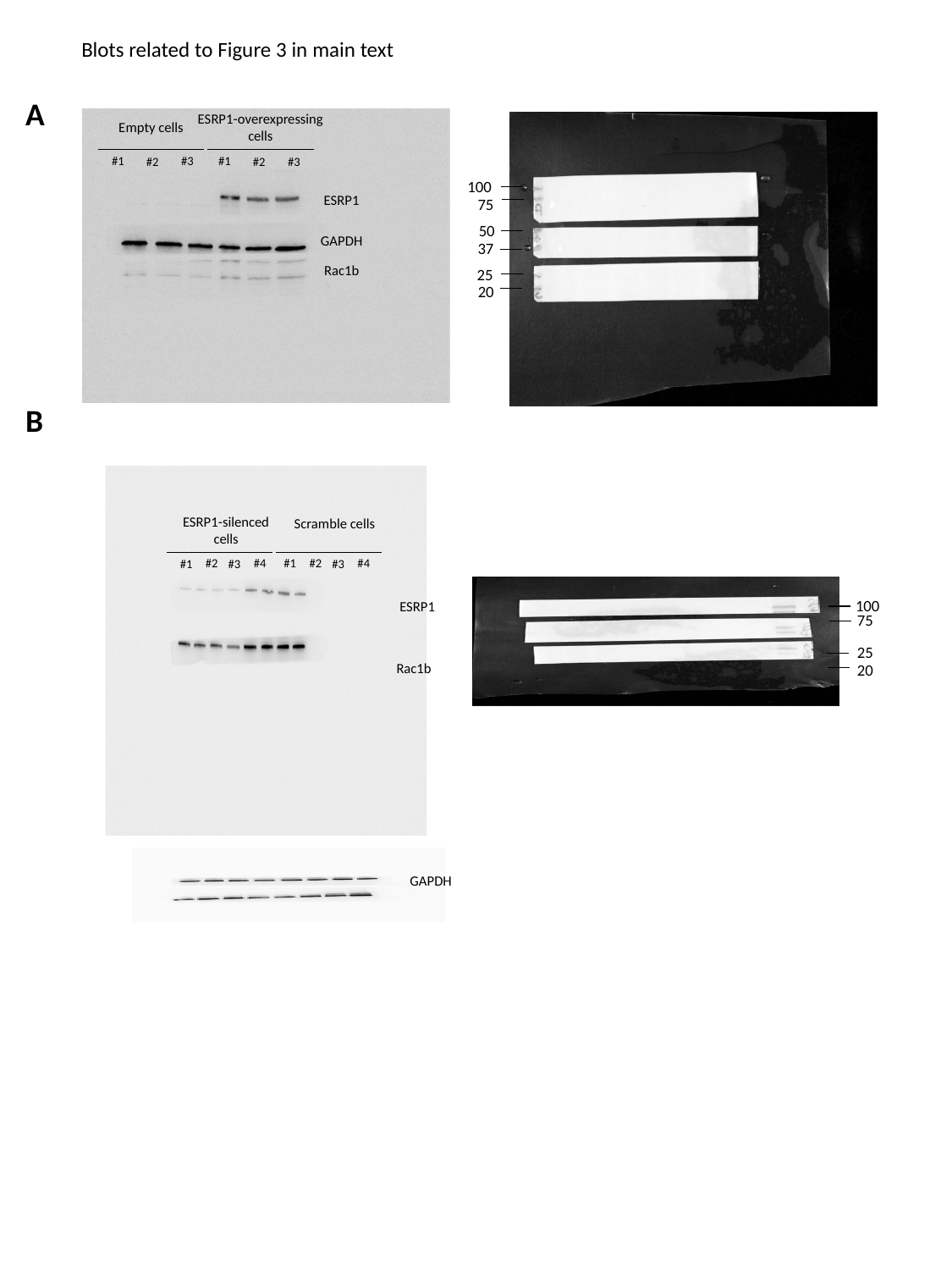

Blots related to Figure 3 in main text
A
ESRP1-overexpressing
cells
Empty cells
#1
#1
#3
#3
#2
#2
100
ESRP1
75
50
GAPDH
37
Rac1b
25
20
B
ESRP1-silenced
cells
Scramble cells
#2
#4
#2
#1
#4
#3
#1
#3
100
ESRP1
75
25
Rac1b
20
GAPDH

## Slide 2
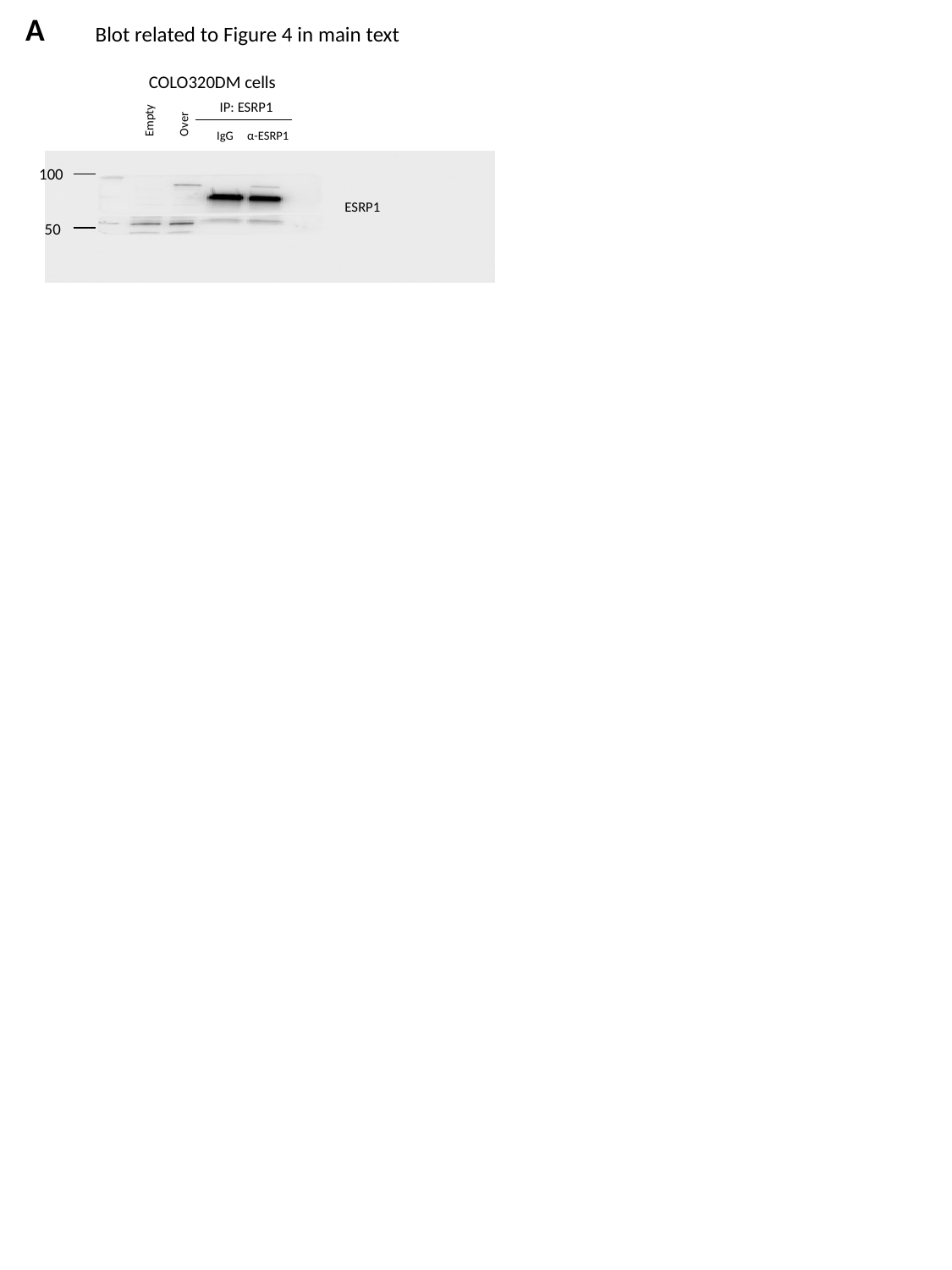

A
Blot related to Figure 4 in main text
COLO320DM cells
IP: ESRP1
Empty
Over
IgG
α-ESRP1
100
ESRP1
50

## Slide 3
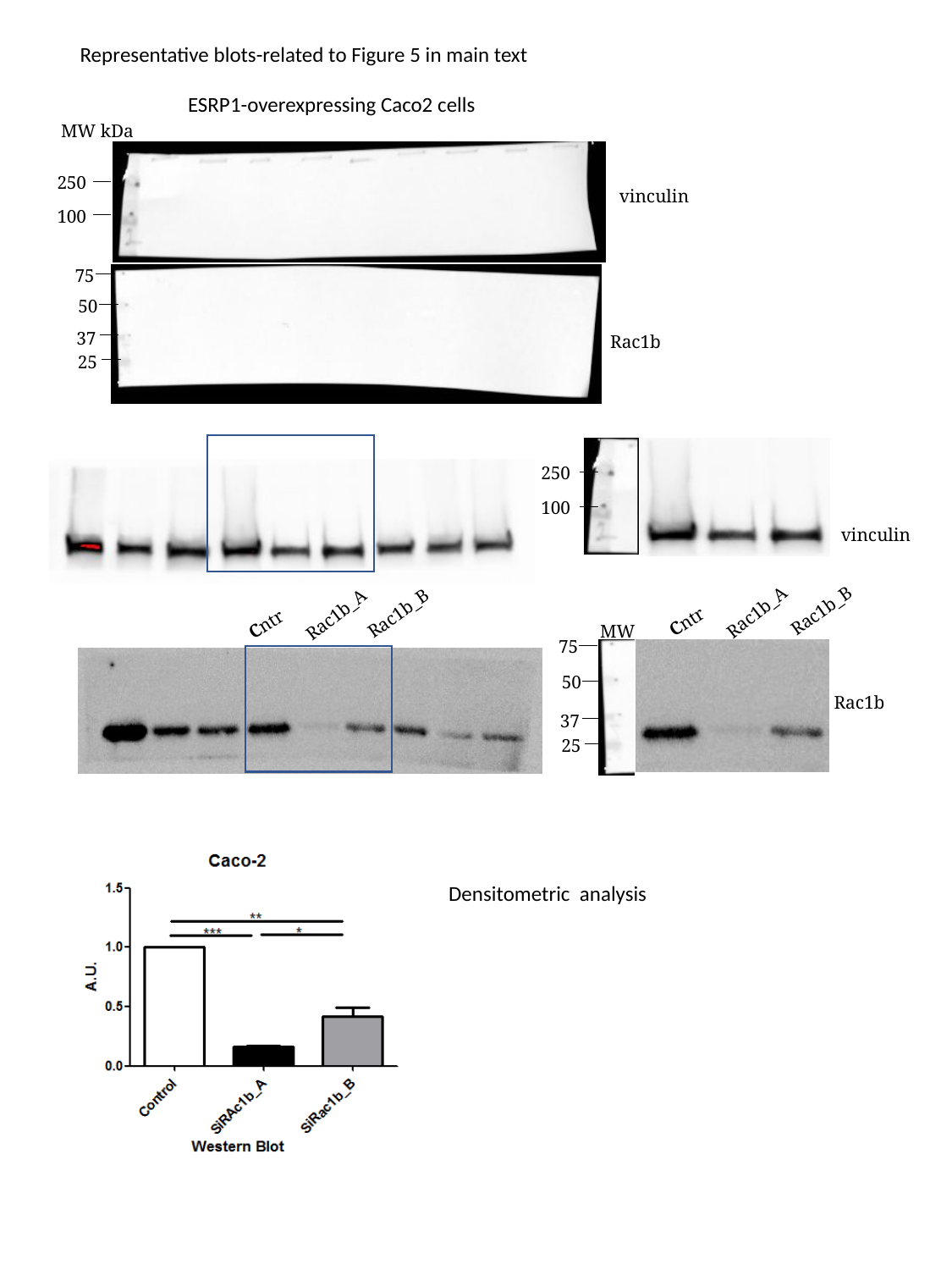

Representative blots-related to Figure 5 in main text
ESRP1-overexpressing Caco2 cells
MW kDa
250
100
75
50
37
25
vinculin
Rac1b
250
100
vinculin
Rac1b_B
Rac1b_A
Cntr
MW
75
50
37
25
Rac1b_B
Rac1b_A
Cntr
Rac1b
Densitometric analysis
